# Top-of-Atmosphere Retrieval of Multiple Crop Traits Using Variational Heteroscedastic Gaussian Processes within a Hybrid Workflow

**DOI:** 10.3390/rs13081589

**Published:** 2021-04-20

**Authors:** José Estévez, Katja Berger, Jorge Vicent, Juan Pablo Rivera-Caicedo, Matthias Wocher, Jochem Verrelst

**Affiliations:** 1 Image Processing Laboratory (IPL), Parc Científic, Universitat de València, 46980 Paterna, Spain; 2 Department of Geography, Ludwig-Maximilians-Universität München (LMU), Luisenstr. 37, 80333 Munich, Germany; 3 Magellium, 31520 Toulouse, France; 4 Secretary of Research and Postgraduate, CONACYT-UAN, Tepic 63155, Mexico

**Keywords:** biophysical and biochemical traits, top-of-atmosphere reflectance, Sentinel-2, variational heteroscedastic Gaussian process regression, hybrid model

## Abstract

In support of cropland monitoring, operational Copernicus Sentinel-2 (S2) data became available globally and can be explored for the retrieval of important crop traits. Based on a hybrid workflow, retrieval models for six essential biochemical and biophysical crop traits were developed for both S2 bottom-of-atmosphere (BOA) L2A and S2 top-of-atmosphere (TOA) L1C data. A variational heteroscedastic Gaussian process regression (VHGPR) algorithm was trained with simulations generated by the combined leaf-canopy reflectance model PROSAILat the BOA scale and further combined with the Second Simulation of a Satellite Signal in the Solar Spectrum (6SV) atmosphere model at the TOA scale. Established VHGPR models were then applied to S2 L1C and L2A reflectance data for mapping: leaf chlorophyll content (*C_ab_*), leaf water content (*C_w_*), fractional vegetation coverage (FVC), leaf area index (LAI), and upscaled leaf biochemical compounds, i.e., LAI * *C_ab_* (laiCab) and LAI * *C_w_* (laiCw). Estimated variables were validated using in situ reference data collected during the Munich-North-Isar field campaigns within growing seasons of maize and winter wheat in the years 2017 and 2018. For leaf biochemicals, retrieval from BOA reflectance slightly outperformed results from TOA reflectance, e.g., obtaining a root mean squared error (RMSE) of 6.5 μg/cm^2^ (BOA) vs. 8 μg/cm^2^ (TOA) in the case of *C_ab_*. For the majority of canopy-level variables, instead, estimation accuracy was higher when using TOA reflectance data, e.g., with an RMSE of 139 g/m^2^ (BOA) vs. 113 g/m^2^ (TOA) for laiCw. Derived maps were further compared against reference products obtained from the ESA Sentinel Application Platform (SNAP) Biophysical Processor. Altogether, the consistency between L1C and L2A retrievals confirmed that crop traits can potentially be estimated directly from TOA reflectance data. Successful mapping of canopy-level crop traits including information about prediction confidence suggests that the models can be transferred over spatial and temporal scales and, therefore, can contribute to decision-making processes for cropland management.

## 1 Introduction

The global agricultural sector faces in 2021 multiple challenges of ensuring sufficient food production for an increasing world population, while at the same time mitigating negative environmental impacts under changing climatic conditions [1,2]. In this context, Earth observation (EO) data have been proven to be a valuable nondestructive basis for spatial and temporal monitoring of crop status and development through the retrieval of vegetation traits. A diversity of agricultural applications use these traits, including yield forecasting, land use monitoring, precision farming, phenotyping activities, and other ecosystem services [2]. Spatial and temporal information of agronomic variables provides comprehensive insights into photosynthetic potential and functioning, and thus the physiological status and health of agricultural crops [3]. The leaf area index (LAI), defined as the total one-sided area of leaf tissue per unit ground surface area, is among the most important vegetation traits to quantify [4,5]. Being a key component of biogeochemical cycles in ecosystems, the LAI drives the canopy microclimate and influences rainfall interception and the amount of intercepted radiation, and thus carbon gas exchange [5,6]. In remote sensing studies, the LAI should be understood as the effective plant area index (PAI) taking into account the nonrandom positions of leaves leading to clumping, as well as the influences of other plant organs, such as stems and fruits, on the optical measurements [7,8]. For simplicity, we use the terms “LAI” and “effective PAI” interchangeably.

The primary light harvesting pigments of chlorophyll a + b content (*C_ab_*) strongly determine the utilization of photosynthetically active radiation (PAR, 400−700 nm) by the crop for the process of photosynthesis. Hence, *C_ab_* provides essential information about the photosynthetic potential [9,10]. As another important trait, water content in plants has a close relation to vegetation transpiration and net primary production [11]. For agricultural crops, the quantification of leaf water content (*C_w_*) and canopy water content (laiCw) is important in view of water use efficiency [12] and the monitoring of plant physiological status, health, and residual moisture during the harvesting process [1,13,14].

For agricultural applications, spatiotemporal explicit information of key crop properties is needed, which can be provided increasingly efficiently, accurately, and precisely by remotely sensed data. Therefore, in the last few decades, an expanding arsenal of optical and thermal satellite sensors has been exploited for the retrieval of vegetation traits across a range of spatial and temporal scales [15]. Among the most auspicious optical EO satellites for the retrieval of vegetation traits currently orbiting the globe are the Copernicus Sentinel families; and in particular, the twin constellation of Sentinel-2A and -2B (S2), dedicated to terrestrial Earth observation. With their relatively short global revisit time of five days (2−3 days in the mid-latitudes), spatial resolutions at 10 m, 20 m, and 60 m, and adequate spectral resolution with 13 bands covering the visible and near-infrared (VNIR, approximately 400−1300 nm) to shortwave infrared (SWIR, approximately 1300−2500 nm) spectral domains, the S2 mission can be seen as an ideal data source for agricultural applications in any corner of the world [16–18]. The S2 constellation together with earlier operational EO satellite missions led to an unprecedented availability of optical data, which in turn stimulated the development of retrieval algorithms in multiple methodological directions: vegetation traits cannot be directly measured by the EO satellite sensors; hence, intermediate models are needed to establish the relationships between the measured spectral signal (i.e., reflectance or radiance) and the variables or traits of interest.

A taxonomy of retrieval methods was provided by Verrelst et al. [19,20], with hybrid approaches evolving as the most promising category. Hybrid methods combine the advantages of machine learning regression algorithms (MLRAs) with radiative transfer models (RTMs), by training MLRAs over (optimally) sampled RTM data bases [21–25]. Merging mechanistic and data-driven methods seems ideal for variable retrieval problems due to the complementary nature: RTMs provide physical constraints and domain knowledge to fast and efficient machine learning algorithms [19]. With respect to MLRA, Gaussian process regression (GPR), introduced by Rasmussen et al. [26], has proven to be one of the most appealing algorithms, delivering highly competitive accuracy [27,28]. As the most interesting feature, GPR provides associated uncertainty of the mean prediction, which can be used as a quality indicator. For instance, if uncertainties obtained by a locally trained model are in the same order as produced over an arbitrary site (without validation data) under different conditions (e.g., spatial and time), we can assume that the model provides same quality for both sites [29]. Hence, this special capability enables the transfer of developed models into space and time, reducing thus the need for time-consuming campaigns to collect reference data for model calibration and validation [29]. Moreover, GPR is simple to train and works well with a relative small data set, as opposed to other methods like neural networks (NNs) [30].

Despite the great advantages of the algorithm, an important challenge in the practical use of GPR when dealing with spectral data is that the signal and noise are usually correlated: the standard formulation assumes that the variance of the noise process *σ_n_* is independent of the signal. This strong assumption of homoscedasticity is generally broken in many EO data-related retrieval problems since the acquisition process is typically affected by noise. In order to deal with input-dependent noise variance, heteroscedastic Gaussian processes were proposed, letting noise power vary smoothly throughout the input space. In this respect, the marginalized variational approximation yields a rich and flexible model [31], named variational heteroscedastic GPR (VHGPR). These models not only showed very good results in biophysical variable retrieval from EO data [32,33], but also proved to slightly outperform standard GPR in terms of accuracy [24,34].

Regarding useful RTMs for hybrid schemes, among the most widely applied RTMs are the leaf optical properties model PROSPECT [35] and Scattering by Arbitrarily Inclined Leaves (SAIL) [36]. Coupling of these models to PROSAIL [37,38] allows upscaling retrieval problems from the leaf to the canopy level. A hybrid retrieval workflow was introduced with the Biophysical Processor through the Sentinel Application Platform (SNAP) [39]. Hereby, NNs were trained over a simulated spectral database generated by the PROSAIL model. The SNAP Biophysical Processor is therefore named “SNAP NN” throughout the manuscript, referring to the neural network-based algorithm. The SNAP NN toolbox provides Level-2B products, such as LAI and fractional vegetation cover (FVC), from S2 reflectance data at both scales (L1C and L2A). Still, comparison studies are needed across diverse canopy types to evaluate the capability of the SNAP NN models [40].

Regardless of the selected method, the majority of studies exploited retrieval techniques by using bottom-of-atmosphere (BOA) reflectance. This is common practice taught in all remote sensing textbooks with the obvious rationale behind it to retrieve correct surface reflectance by removing atmospheric effects. Consequently, the variability of the received signal is only driven by the biochemical and biophysical properties of the vegetated surfaces. As an alternative approach, a few studies attempted to infer variables of interest from top-of-atmosphere (TOA) reflectance or radiance [41–51]. Exploiting TOA radiance data directly for retrieval has the advantage of avoiding critical atmospheric correction, where potential errors can be passed to subsequent retrieval processes [43]. For instance, measurements of diverse atmospheric properties, such as aerosols or water vapor content, are often only available within a time shift or with geolocation mismatch, which strongly increases the uncertainty of the correction process [52]. However, a sound understanding of the atmospheric processes is required to ensure a successful retrieval from TOA data. To achieve this, TOA retrieval methods often relied on the coupling of a vegetation RTM with an atmosphere RTM [34,47,50,51]. Atmosphere RTMs explicitly model the atmospheric effects on the radiance emitted by a surface. Hence, the interaction of radiation with the atmosphere is calculated, accounting for different gaseous absorptions assuming anisotropic or Lambertian surfaces [53]. Among the most widespread atmospheric models are MODerate resolution atmospheric TRANsmission (MODTRAN) [54], Second Simulation of a Satellite Signal in the Solar Spectrum (6SV) [55], and libRadtran [56].

Recent TOA retrieval attempts relied on the coupled PROSAIL-6SV models and the Lambertian assumption, which likewise led to promising LAI mapping results [34,50]. Both studies demonstrated the potential of vegetation properties’ estimation from TOA radiance data; theoretically by means of a global sensitivity analysis [50] (GSA) and practically with an LAI case study using S2 data [34]. Here, the hybrid retrieval strategy relied on VHGPR algorithms trained over simulations from PROSAIL at the canopy scale and from PROSAIL-6SV models at the atmosphere scale. These RTMs were chosen because of their simplicity and public availability. For instance, the leaf and canopy RTMs are implemented in the Automated Radiative Transfer Models Operator (ARTMO [57]), and the 6SV code(6SV 2.1) [58,59] is inserted within the Atmospheric Look-up table Generator (ALG [60]) software frameworks. Trained models were then applied to S2 L2A (BOA) and L1C (TOA) data for LAI mapping and validation with field measurements. With this, the study of Estévez et al. [34] demonstrated obvious benefits of the developed method, such as the fast mapping and provision of uncertainty estimates. However, the transferability of this approach to other vegetation traits still remains to be investigated.

In light of the above, the main objective of this work was to develop a hybrid retrieval workflow for the estimation of multiple vegetation traits from S2 TOA reflectance data. As a first sub-objective, it was aimed to derive crop traits from BOA reflectance in order to assess the accuracy, uncertainty, and consistency between both BOA- and TOA-retrieved vegetation products. As a second sub-objective, the produced maps by the proposed hybrid workflow were subsequently compared against reference biophysical products generated by the SNAP NN Biophysical Processor toolbox.

## 2 Materials and Methods

### 2.1 Experimental Site and Satellite Data

**Figure 1:**
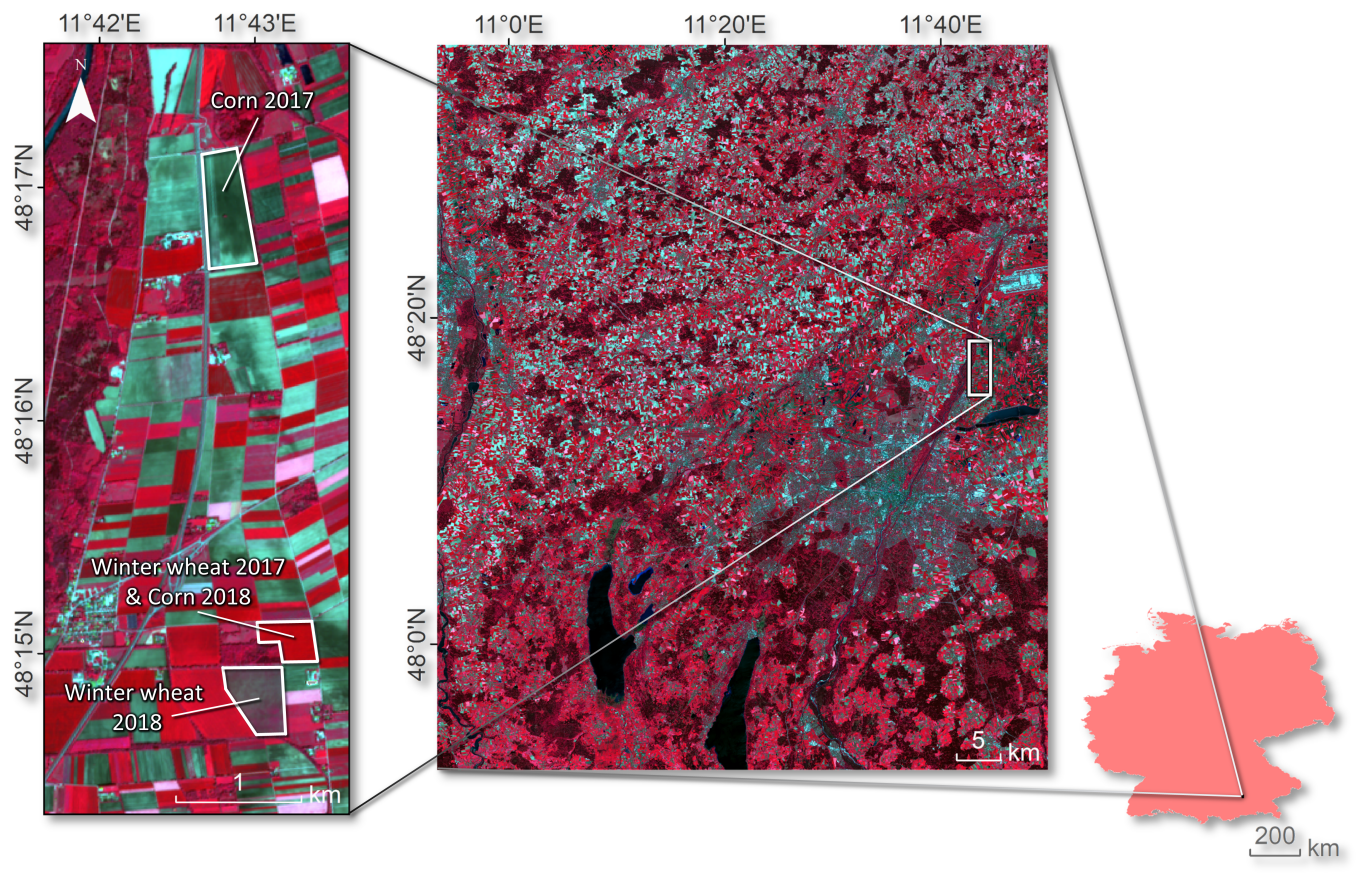
Munich-North-Isar (MNI) test sites of maize (corn) and winter wheat rotation in 2017 and 2018: Sentinel-2A (S2) RGB (R: B8, G: B4, B: B3) from 17/05/2017 (left); MNI location within Bavaria and Germany (lower right).

The data base exploited for our study was provided from extensive field measurements at a test site in the North of Munich, in Southern Germany: the Munich-North-Isar (MNI) campaigns (N 48°16′, E 11°42′). The test site is located east of the river Isar and belongs to the communal farmland of the city of Munich (see Figure 1). During the growing periods of 2017 and 2018, field spectroscopic, destructive, and non-destructive measurements of biochemical and biophysical crop characteristics were carried out on winter wheat (*Triticum aestivum*) and maize (Zea *maize*). In the fields, nine elementary sampling units (ESUs) of 10 × 10 m were defined (see the figure in Berger et al. [24]), delineating an area of 30 × 30 m, which aimed to correspond to the spatial extent of a pixel of the future Environmental Mapping and Analysis Program (EnMAP) [61]. Leaf area index measurements, in m^2^/m^2^, were performed using an LI-COR Biosciences LAI-2200 device, equipped with a GPS sensor. A few LAI measurements of very mature winter wheat growth stages were excluded for validation (i.e., LAI > 5), due to the strong influence of non-photosynthetic plant tissues on the sensor, leading to apparently high LAI values. Measurements with this instrument may lead to LAI overestimation due to radiation interception of yellow leaves, stems, and heads (ears), as found by Jovanovic and Annandale [62] for triticale and rye. Leaf chlorophyll content was sampled with a Konica-Minolta SPAD-502 handheld instrument from five leaves at different plant heights per ESU and was then averaged to obtain a representative 30 × 30 m mean value in μg/cm^2^. The device was calibrated in a preceding field campaign against destructive measurements of *C_ab_* at different crop growth stages [63]. For leaf water content, two leaves were destructively and randomly sampled within each of the defined ESUs (18 samples per date). Leaf samples were weighed, packed in bags, and brought to the lab. There, leaf area was measured using an LI-COR Biosciences LI-3000C scanner attached to the LI-3050C conveyor belt accessory. The final *C_w_* in cm was obtained from the mass difference of sample leaves per unit leaf area before and after oven-drying at 105 °C (minimum of 24 h) to constant weight [14,64]. Additionally, measured leaf biochemicals were upscaled to the canopy level by multiplication with LAI, resulting in canopy chlorophyll content, i.e., LAI *C_ab_ (laiCab), and canopy water content, i.e., LAI * *C_w_* (laiCw), all in g/m^2^. Table 1 gives an overview of all measured and calculated variables with mean values and ranges for each acquisition date.

Google Earth Engine (GEE), a cloud computing platform designed for geo-spatial analysis at the petabyte scale [65], was used to obtain all available S2 L1C and corresponding L2A images (maximum 1% cloud cover) within the vegetation growing seasons of 2017 and 2018 (between 15 March and 30 September) over the MNI test site. The GEE catalog was found to be an optimal data source for the purposes of this study; it is continuously updated, ingesting data from different archives, among others the ESA Copernicus Open Access Hub. The dates of the acquired S2 images with the corresponding dates of the MNI campaigns are indicated along with the measurement values of crop traits in Table 1. BOA and TOA reflectance were extracted from the scenes in order to match the pseudo-EnMAP pixel average of the in situ data collections. Note that the moderate total number of only 14 in situ measurement points (from approximately 30 for the two seasons) was either due to the temporal mismatch of S2 scenes and field reference data or caused by local weather conditions: the average cloud cover over the MNI site region is around 5.5 okta [63]. Despite the limited number of samples, this should nevertheless be an adequate number to compare the retrieval performances between the BOA and TOA scales.

### 2.2 Theory, Models, and Retrieval Strategy

**Figure 2:**
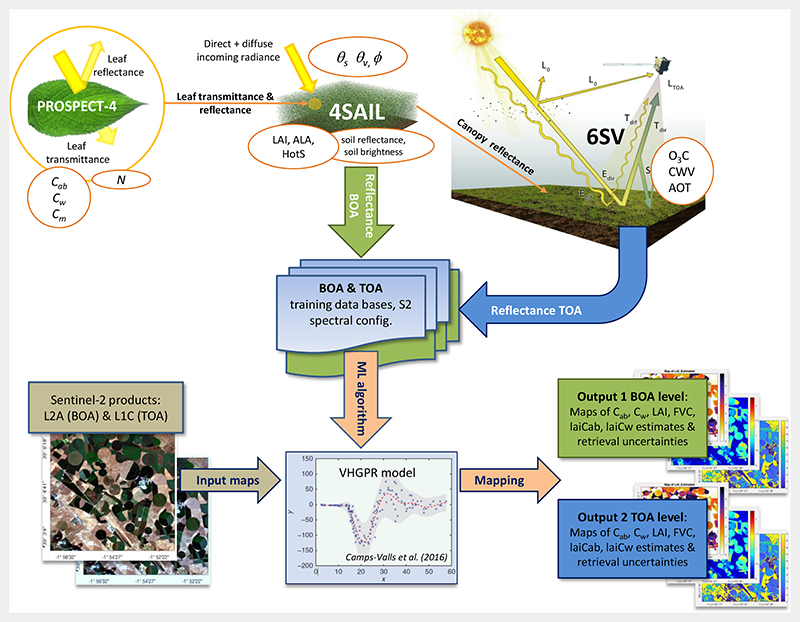
Flowchart of the pursued workflow. Top: coupling of leaf-canopy-atmosphere radiative transfer models (RTMs) for the generation of the data base for training. Bottom: training of variational heteroscedastic Gaussian process regression (VHGPR) for vegetation traits mapping at S2 bottom-of-atmosphere (BOA) and TOA scales. See also Table 2 for the explanations of the model input parameters. Exemplary S2 images used from Barrax campaigns [34]. ALA, average leaf angle; HotS, hot spot parameter; 6SV, Second Simulation of a Satellite Signal in the Solar Spectrum; CWV, columnar water vapor; AOT, Aerosol optical thickness; FVC, fractional vegetation coverage.

A hybrid retrieval workflow was adopted from two earlier exploratory TOA retrieval studies [34,50]. The conceptual overview of the workflow is provided in Figure 2. Its key steps are detailed in the following sections, starting with a brief description of the top-of-canopy (TOC) model and simulations, followed by top-of-atmosphere radiative transfer modeling, the VHGPR algorithm used functioning as core retrieval model, and the outline of the workflow. Finally, the comparison exercise with the SNAP Biophysical Processor is described.

#### 2.2.1 Top-of-Canopy Radiative Transfer Modeling

At the BOA scale, a wide range of vegetation states was assumed to simulate the corresponding TOC reflectance with the PROSAIL model. Table 2 lists the biochemical and biophysical input parameters with the applied units, ranges, and distributions. We employed an older version of the leaf optical properties model series (PROSPECT-4 [35]), since the total number of input parameters should be kept small. The additional input parameters in the more recent PROSPECT versions, such as carotenoids or anthocyanins, were not of interest for this research, but may be explored in a future study. However, in contrast to the study of Estévez et al. [34], here, all PROSPECT-4 input parameter were ranged, and also, fractional vegetation cover (FVC) was added. In SAIL, the canopy is considered as a 1D homogeneous structure, which means that the variations of the macroscopic properties in the horizontal plane, as well as the clumping of canopy elements are neglected. FVC is therefore approximated empirically from the gap fraction at nadir. The gap fraction can be expressed mathematically as *P* = *exp*(−*k* × *LAI*), where *k* is the extinction coefficient [66]. In SAIL, *k* is calculated based on the leaf inclination distribution and the viewing angle [67]. Further, to determine the estimation accuracy of biochemical variables at the canopy level, *C_ab_* and *C_w_* were multiplied with the LAI to obtain laiCab and laiCw, in g/m^2^.

Based on possible PROSAIL input parameter ranges according to Table 2, a random subset of 1000 combinations was used to simulate the bi-directional reflectance of vegetated canopies. This size was small compared to the typical sampling sizes applied within radiometric look-up table (LUT) inversion strategies, as well as compared to training data sets for neural networks [39,68]. However, a standard implementation of a GPR typically cannot cope with more than a few thousand samples within a reasonable computational time. This apparent limitation is well compensated by the algorithms, which require only relatively small training data sets and can adopt very flexible kernel functions for establishing nonlinear relationships between spectral observations and variables of interest [32].

To address uncertainties associated with sensor measurement accuracy or data processing including radiometric calibration, atmospheric and geometric corrections, as well as the limited realism of the RTM with respect to surface heterogeneity or failure in parameterization, the inclusion of noise may be considered [21,64,69,70].

White Gaussian noise was applied to the simulated spectra, according to the noise model provided in Equation (1)[39]:

(1) $$R^{*}(\lambda )=R(\lambda )\cdot \left(1+\frac{MD(\lambda )+MI}{100}\right)+AD(\lambda )+AI$$

where *R(λ)* and R* (λ) represent the raw simulated reflectance for band *λ* and the reflectance with uncertainties for band *λ*, respectively. MD denotes the multiplicative wavelength-dependent noise, MI the multiplicative wavelength-independent noise. AD and AI stand for the additive wavelength-dependent and -independent noises, respectively. According to internal tests, we applied AD, AI = 0.01 and MD, MI = 4% for all the bands. Similar noise levels were introduced to optimize LUT-based inversion for LAI retrieval [71–73] or to generate training data for machine learning regression algorithms [21,50].

The final data base of the combined PROSAIL input variables and TOC reflectance output was subsequently used for the development of the retrieval models at the BOA (S2 L2A) scale.

#### 2.2.2 Top-of-Atmosphere Radiative Transfer Modeling

At the TOA scale, atmospheric reflectance was simulated with the 6SV model. For this, we selected mid-latitude summer atmospheric profile mode. In Table 2, the details of the atmospheric model input parameters and their respective ranges can be found. The outputs of 6SV were the following atmospheric transfer functions for each combination of the key input variables; see also Vicent et al. [60]:

- Intrinsic atmospheric reflectance (*ρ*_0_),
- Total gas transmittance (*T_gas_*),
- Total downwards and upwards transmittance due to scattering (*T_dwn_* and *T_up_*),
- Spherical albedo (*S*), which denotes the atmospheric reflectance spectrum for the photons backscattered to the surface (*S*), and
- Extraterrestrial solar irradiance (*I*_0_) in mW·m^−2^·nm^−1^.

TOA radiance spectra (*L*) were then calculated by coupling the generated atmospheric transfer functions from 6SV with the Lambertian surface reflectance (*ρ*) from PROSAIL following the equation:

(2) $$L=\frac{I_{0}\mu_{il}}{\pi }T_{gas}\left[\rho_{0}+\frac{T_{dwn}T_{up}\rho }{1-S\rho }\right]\equiv \frac{I_{0}\mu_{il}}{\pi }\rho_{toa}$$

where *μ_il_* = cos *θ_il_*, with *θ_il_* being the solar zenith angle. The magnitude *ρ_toa_* is the TOA reflectance (i.e., Sentinel-2 L1C product), which can be understood as the TOA radiance normalized by the solar irradiance. For the sake of simplicity, the spectral dependency of all terms within Equation (2) was omitted.

For the upscaling from BOA to TOA, it was necessary to resample the 1 nm PROSAIL spectral resolution to the 2.5 nm sampling of 6SV atmospheric simulations using spline interpolation. With this common spectral resampling method, both data sets generated by PROSAIL and 6SV were randomly combined, and a total number of 1000 samples was propagated into TOA radiance, following Equation (2).

The TOC-to-TOA coupling was performed by means of ARTMO’s TOC2TOA toolbox, which can generate the final TOA table consisting of pairs of radiance (or reflectance) spectra with associated vegetation properties and atmosphere parameters [50]. As opposed to the earlier version presented in Estévez et al. [34], two novelties were introduced in the updated toolbox (v. 1.02): (i) with the inclusion of Equation (2), both TOA radiance and reflectance are provided; and (ii) viewing and observation geometry can be ranged. This was now possible through allowing a buffer around each geometry value, even when random numbers were introduced into the TOC or atmospheric geometry. Instead, in the earlier TOC2TOA version, it was only possible to synchronize the TOC and atmospheric simulations with a fixed geometry. In the actual tool, two training data sets generated with random geometry values can be coupled. The conversion of TOA radiance into the S2 spectral configuration was carried out by convolving the full spectra to the resolution of S2 using built-in spectral response functions (SRFs). The information of the SRFs was obtained from ESA’s website [74] (accessed 14 April 2021).

#### 2.2.3 Variational Heteroscedastic Gaussian Process Regression

The main retrieval algorithm of our study relied on the principles of GPR. However, a non-standard variational approximation that allows accurate inference in signaldependent noise scenarios (i.e., under input-dependent noise conditions) was adapted, called VHGPR [31,32]. This marginalized variational approximation renders (approximate) Bayesian inference in the model fast and accurate, providing an analytical expression for the Kullback−Leibler divergence between a proposed distribution and the true posterior distribution. By minimizing this quantity with respect to the proposal distribution, as well as the hyperparameters, accurate estimation of the true posterior can be obtained while concurrently performing model selection. The expression of the approximate mean and variance of the posterior can then be computed in closed form. Variational techniques allow approximating intractable integrals arising in Bayesian inference and machine learning in general. For instance, these techniques are used to provide analytical approximations to the posterior probability of the unobserved variables and, hence, apply statistical inference over these variables [32]. Detailed descriptions of the VHGPR algorithms including mathematical expressions and equations were provided by Lázaro-Gredilla and Titsias [31] and Lázaro-Gredilla [32].

#### 2.2.4 Delineation of the Hybrid Workflow

In summary, the retrieval workflow (see also Figure 2) consisted of the following main steps:

1. generation of training data bases with the models PROSAIL and 6SV and coupling for upscaling at the TOA using atmospheric transfer functions;
2. training the VHGPR algorithm over the simulated data bases to establish variablespecific retrieval models for both scales;
3. validation with in situ field measurements from the MNI site; and
4. mapping multiple crop traits and corresponding uncertainties using S2 scenes from a selected date.

For all spectral data, ten out of the 13 available S2 bands were employed or established using S2-SRFs, covering 10−20 m pixel sizes with S2 central wavelengths of 493 nm, 560 nm, 665 nm, 704 nm, 740 nm, 783 nm, 833 nm, 865 nm, 1610 nm, and 2190 nm. In Estévez et al. [34], the contribution of these bands was analyzed for LAI retrieval. Since the validation results showed insignificant differences when using eight, nine, or ten bands, no large impact was assumed. Hence, it was decided to keep all ten bands for further processing to assure a maximum of spectral information that may be required for the retrieval of multiple crop traits. The three bands of a 60 m pixel size (443 nm, 945 nm, and 1374 nm) were excluded since their focus was on cloud screening and atmospheric corrections. Finally, images were resampled to a 10 m ground sampling distance.

It must also be noted that PROSAIL is a vegetation canopy model and not prepared to simulate the spectral variability of non-vegetated surfaces. Hence, around 30 distinct nonvegetated samples (e.g., soil, man-made surfaces, water bodies) were visually identified from S2 L2A and L1C products and added to the training data bases (TOC and TOA).

The TOC and TOA tables were used to train the VHGPR algorithm for the generation of variable-specific retrieval models applicable to S2 L2A and L1C data. With the exception of FVC, models were only established for those vegetation traits where validation data were available. When training an MLRA with simulated data, a first requirement is to evaluate the theoretical performances of the established models, i.e., the accuracy using simulated data. To do so, a k-fold cross-validation sampling scheme was applied, in which the training data set was split into five subsets (k = 5). Finally, in order to assess the capability of the VHGPR models to generate maps of multiple crop traits, the retrieval models were applied to estimate actual *C_ab_*, *C_w_*, FVC, LAI, laiCab, and laiCw using ARTMO’s MLRA toolbox [75]. Second, a cloud-free spatial subset of S2 L1C (TOA) and L2A (BOA) imagery from 6 July 2017 was chosen to evaluate mapping accuracy at both scales.

#### 2.2.5 Comparison against SNAP Biophysical Processor Vegetation Products

Since a limited number of in situ samples may not be conclusive enough to assess the suitability of the developed VHGPR models, an additional comparison exercise was performed using the Biophysical Processor toolbox from SNAP (v.6.0.0) [39]. Among others, the processor provides the following vegetation products: LAI, laiCab, laiCw, and FVC from L1C (TOA) and L2A (BOA) data. The rationale for comparing the results of the here presented hybrid retrieval workflow with these products lied in the fact that the Biophysical Processor can be considered as a benchmark product used by an increasing number of studies and image processing applications [40,73,76,77]. Hence, a direct comparison against the SNAP vegetation products through the computation of scatter plots and relative error maps gave a quantitative assessment of the models’ performance across all land cover types present around the test sites.

Lastly, to streamline the whole analysis for all the conducted validation including BOA and TOA comparison, common goodness-of-fit statistics, i.e., the root mean squared error (RMSE) in variable-specific units, normalized RMSE (NRMSE in %, being the RMSE divided by the range of observations), and coefficient of determination (R^2^), are given.

## 3 Results

### 3.1 Theoretical Results of the VHGPR Models

The theoretical goodness-of-fit results, displayed in Table 3, revealed the following trends: (1) there were generally small, but statistically non-significant differences between the BOA and TOA results; (2) the good performance of LAI estimation benefited from the accuracy of upscaled leaf variables (i.e., for laiCab and laiCw). All variables reached reasonable performances, with laiCab, laiCw, and FVC obtaining relative errors (i.e., NRMSE) far below 10%. Nevertheless, these statistics merely gave information about theoretical model performances, which were required to assess the reliability of the models’ parameterization. Evaluation against in situ-collected ground validation data would additionally indicate the suitability and portability of the developed models.

### 3.2 Validation against In Situ Data

**Figure 3:**
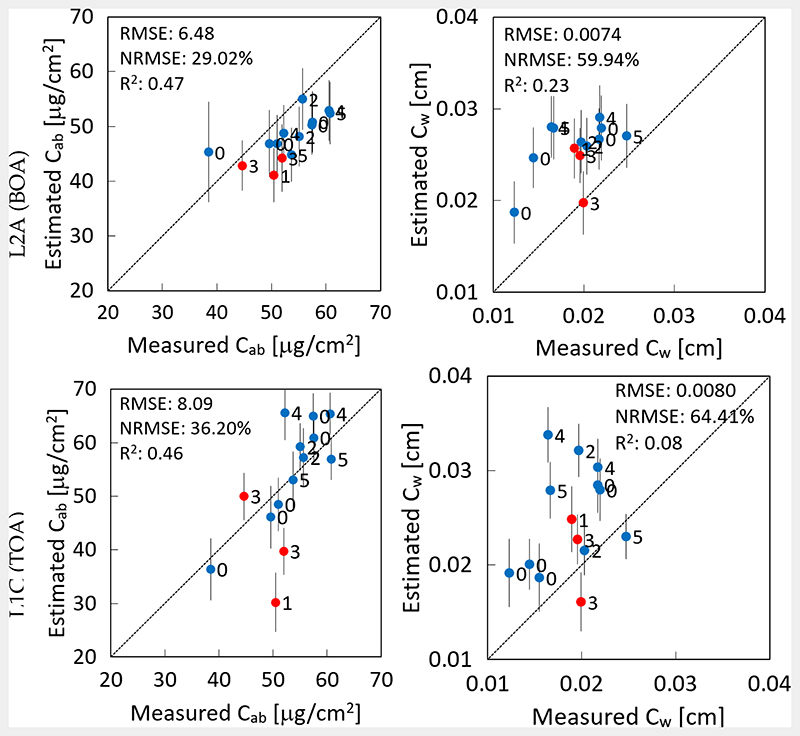
Ground validation of maize and winter wheat over the MNI site by the VHGPR model for retrieval from S2-L2A (BOA) (top) and S2-L1C (TOA) reflectance (bottom). Measured vs. estimated values along the 1:1-line with associated confidence intervals (1 SD) for leaf traits: leaf chlorophyll content, *C_ab_* (left), and leaf water content, *C_w_* (right). Labels indicate the time-shift between the date of in situ collection and S2 acquisition. Vertical bars indicate associated uncertainty estimates.

The VHGPR models’ performance was evaluated against the in situ data set of the MNI campaigns. Hereby, the accuracy of the *C_ab_* and *C_w_* simulations from L2A BOA and from L1C TOA are presented in Figure 3, top and bottom, respectively. Generally, the retrieval from BOA reflectance slightly outperformed the results from TOA reflectance, for both leaf-level variables. The accuracy of *C_ab_* can be considered as good, with the RMSE between 6.5 and 8 μg/cm^2^, though a slight underestimation occurred at the BOA scale. In the case of *C_w_*, overestimation at both scales could be detected, and the retrieval performance was only moderate with relative errors around 60%. Hereby, water loss during transport to the laboratory may play a role, limiting the credibility of the ground-sampled data (see also the Discussion Section).

**Figure 4:**
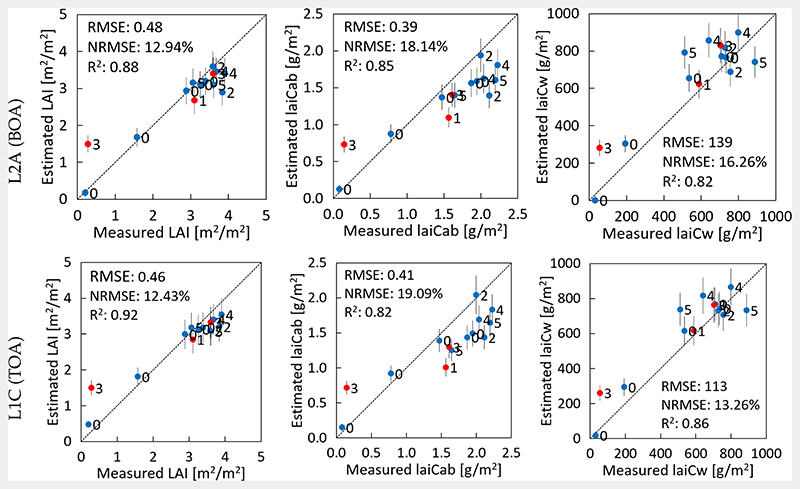
Ground validation of maize and winter wheat over the MNI site by the VHGPR model for retrieval from S2-L2A (BOA) (top) and S2-L1C (TOA) reflectance (bottom). Measured vs. estimated values along the 1:1-line with associated confidence intervals (1 SD) for several canopy variables: LAI (left), canopy chlorophyll content, laiCab (center), and canopy water content, laiCw (right). Labels indicate the time-shift between the date of in situ collection and S2 acquisition. Vertical bars indicate associated uncertainty estimates.

Results for the canopy-level variables against in situ are demonstrated in Figure 4 with retrieval from L2A BOA reflectance in the top plots and from L1C TOA reflectance in the bottom plots. In general, the estimation performance at the canopy level was reasonably accurate, with higher accuracy from the TOA scale for two of the three variables. LAI estimation from TOA reflectance only slightly outperformed BOA estimations (RMSE of 0.46 m^2^/m^2^ versus 0.48 m^2^/m^2^). For laiCab, underestimation occurred, which was related to the underestimation at the leaf scale. Noteworthy is the higher accuracy of laiCw predicted from TOA compared to BOA reflectance, with an NRMSE of 13% vs. 16%. Still, the model slightly overestimated the laiCw values at both scales. Yet, mapping results were needed to inspect these trends at the landscape scale.

As a side remark, the labels in Figures 3 and 4 indicate the time-shift of zero to a maximum of five days between the dates of in situ collection and the corresponding S2 acquisition. However, no clear trend can be identified: even if the data were recorded with an offset of some days, the accuracy was alike compared to the simultaneous recordings. This confirmed the reasonable choice of this time-window. Vertical bars indicate the associated uncertainty intervals for each estimate corresponding to the standard deviation (SD). It can be observed that all bars fall within the same range without the occurrence of outliers.

### 3.3 Mapping Biochemical and Biophysical Crop Traits

**Figure 5:**
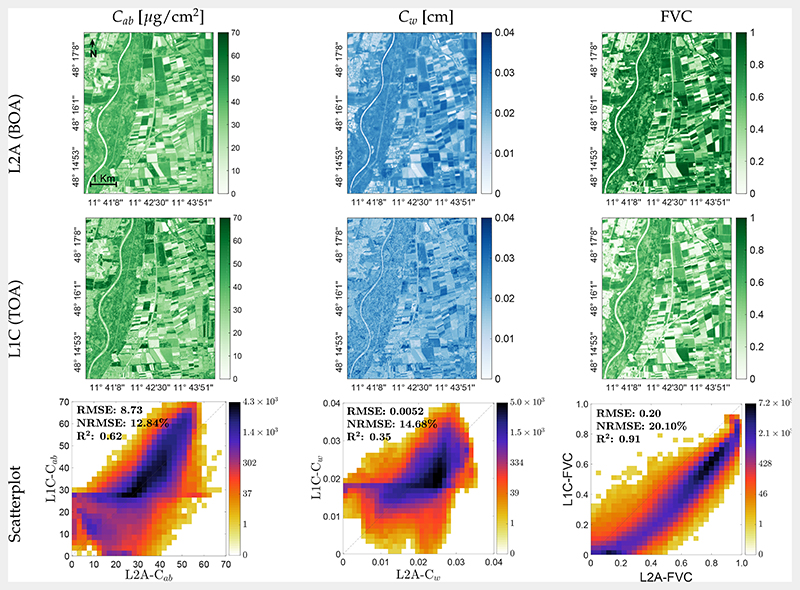
Maps of biochemical leaf traits (mean estimates; *μ*): *C_ab_* (left) and *C_w_* (center), as well as FVC (right), as generated by the VHGPR algorithm from L2A (top) and L1C (middle) data for the MNI test site on 6 July 2017. Scatter plots of both maps with gridded color density (bottom).

**Figure 6:**
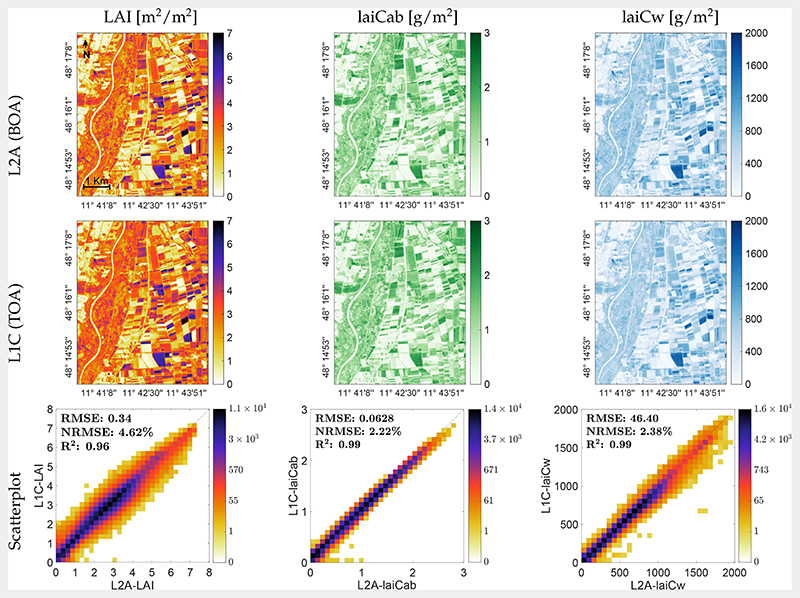
Maps of canopy traits (mean estimates): LAI (left), laiCab (center), and laiCw (right), as generated by the VHGPR algorithm from L2A (top) and L1C (middle) data at the MNI test site on 6 July 2017. Scatter plots of both maps with gridded color density (bottom).

The developed VHGPR models were subsequently applied to the S2 scene covering the MNI test site on 6 July 2017 to map biochemical leaf compounds (*C_ab_* and *C_w_*) and FVC (Figure 5), as well as the LAI with upscaled crop traits (laiCab and laiCw; Figure 6). On this date, both winter wheat and maize fields used for validation (Section 3.2) showed mean LAI values around 3 m^2^/m^2^. While the maize field was fully green, winter wheat started senescence, as indicated by lower estimated within-field values of *C_ab_* around *C_ab_* = 60 μg/cm^2^ versus *C_ab_* = 20 μg/cm^2^ for maize and winter wheat, respectively. In general, for this agricultural area located east of the river Isar, the maps nicely reflected the actual variability of vegetation properties to this point of time, with fully green or mature crops (maize, cereals, rape, and potatoes), but also fallow lands. Scatter plots between BOA and TOA scale estimates are also presented. For the leaf scale (Figure 5, bottom), only moderate correlations were achieved with R^2^ between 0.62 (*C_ab_*) and 0.35 (*C_w_*). Scatter plots suggest a generally broad estimation range. At the canopy level, a significantly higher consistency between the mapping scales was achieved, with R^2^ = 0.91 for FVC and R^2^ around 0.96−0.99 for LAI, laiCab, and laiCw (Figure 6, bottom).

**Figure 7:**
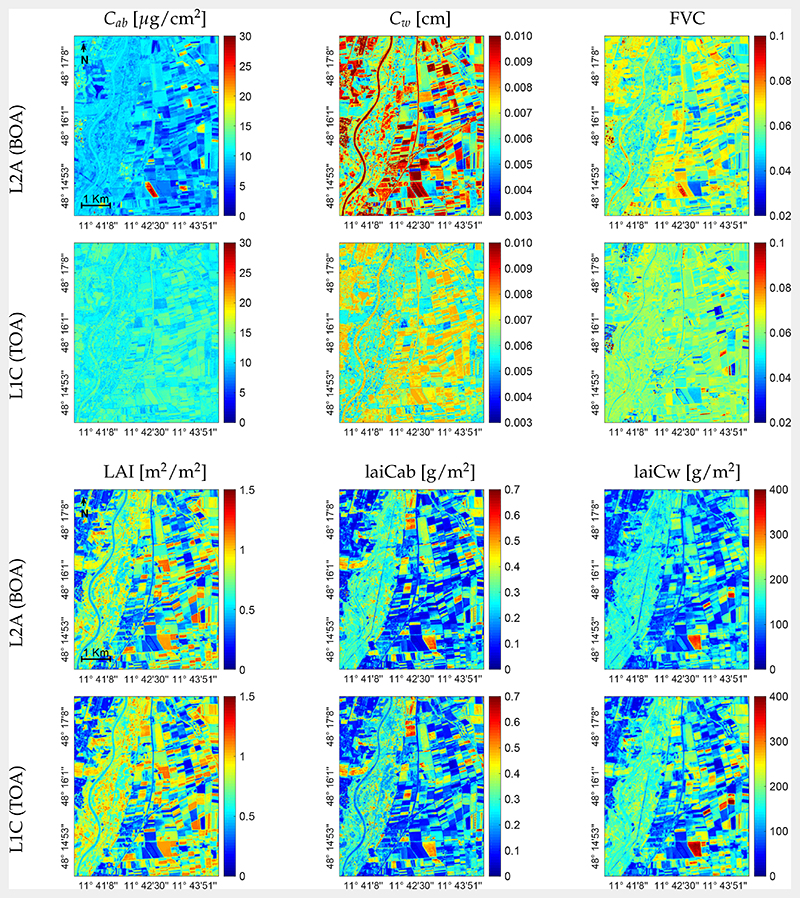
Associated uncertainties (expressed as the standard deviation (SD) around the *μ*) for leaf biochemicals and FVC (top) and canopy crop traits (bottom), generated by the VHGPR algorithm from L2A and L1C data for the MNI test site on 6 July 2017.

In Gaussian process models, a probability distribution over all possible values of the variable of interest is provided (see also Section 2.2.3). The target output (crop trait) is thus given along with a quantification of prediction uncertainty, allowing assessing the VHGPR model retrieval performance over the entire image. Maps of absolute associated uncertainty (expressed as SD around the mean) are provided for all predicted crop traits in Figure 7. Although not shown for brevity, also relative uncertainty (expressed as the coefficient of variation (CV), i.e., the SD divided by the mean) maps were processed. For *C_ab_*, the absolute uncertainty at the BOA scale (Figure 7, top left) revealed mainly values between 5 and 10 μg/cm^2^ over the whole area. There were some fields characterized by high uncertainty, which can be explained by high vegetation coverage: the magnitude of uncertainty values was often highly related to the mean estimates. One field stood out in the BOA-scale *C_ab_* map exposing very high uncertainty (>30 μg/cm^2^). As indicated by all other crop trait estimates, in particular LAI (see Figure 6, top left), this field had very low or non-green vegetation coverage. Though, at the BOA scale, very high *C_ab_* (Figure 5, top left) was estimated (around 70 μg/cm^2^), which appeared out of range. Most likely, this field was a senescent wheat field or fallow land with a specific unknown reflectance signature. Hence, the algorithm failed, which was expressed by a high scale of absolute uncertainty. Compared to BOA retrievals, patterns of uncertainty of leaf compounds appeared more evenly distributed at the TOA scale, with values in a narrower range, thus indicating higher confidence of the estimates.

Absolute uncertainty patterns of the canopy-level traits responded much more alike at both the TOA-L1C and BOA-L2A scales (Figure 7, bottom). Here also, higher estimates were closely related to higher absolute uncertainty. Goodness-of-fit comparisons of absolute and relative uncertainty mapping between the BOA and TOA scales is provided for all crop traits in Table 4. Essentially, we can observe that the canopy variables were more alike between both scales than the leaf variables and that absolute uncertainty patterns (SD) gave more consistent statistics than relative uncertainty (CV).

### 3.4 Comparison against SNAP Vegetation Products

**Figure 8:**
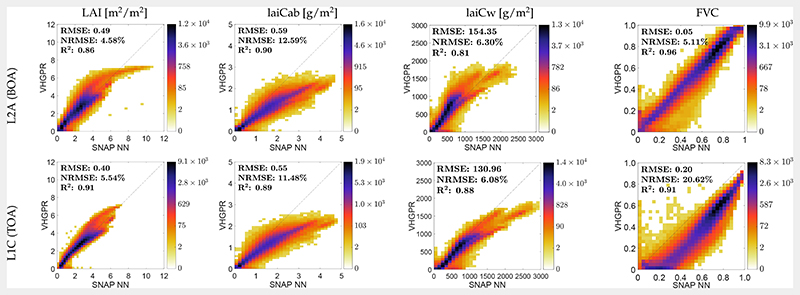
Scatter plots between VHGPR and SNAP NN estimations from L2A (BOA) data (top) and L1C (TOA) data (bottom) for LAI, laiCab, laiCw, and FVC over the MNI site on 6 July 2017.

**Figure 9:**
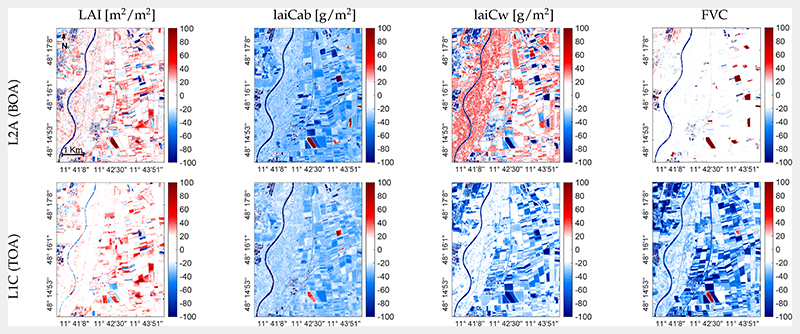
Relative error maps between VHGPR and SNAP NN estimations from L2A (BOA) data (top) and L1C (TOA) data (bottom) for LAI, laiCab, laiCw, and FVC over the MNI site on 6 July 2017.

Since the validation exercise with in situ data coming from two crop types may not be conclusive enough to assess the validity of models that pretend to be generally applicable, our maps were additionally compared against those obtained by the SNAP Biophysical Processor products. With SNAP NN, only biophysical canopy variables were generated, i.e., LAI, laiCab, laiCw, and FVC. For each variable, a scatter plot against the corresponding estimates over the whole scene as obtained by our VHGPR model indicated the retrieval consistency of the respective estimated variable (Figure 8). Between the two products, the high consistency up to LAI = 7 can be observed. The SNAP NN model, however, strongly overestimated the actual LAI values of agricultural fields in the area at this point of time (i.e., with LAI up to 11 at the BOA scale). In the case of laiCab, these differences between the TOA and BOA scales could no longer be observed: in contrast to the VHGPR models, the SNAP NN model systematically overestimated laiCab at both scales. Similar trends can be observed for laiCw and FVC, with an even stronger tendency of SNAP NN model overestimation at the TOA scales. Highest consistency between SNAP NN and the VHGPR models was achieved for LAI retrievals at the TOA scale and FVC estimations at the BOA scale. The strongest discrepancies appeared for higher laiCab (>2 g/m^2^) and laiCw (>1000 g/m^2^) values. Spatial discrepancies can probably be better observed in the relative error maps (Figure 9); blue colors indicate an underestimation of the VHGPR model relative to SNAP NN. Remarkably, hereby is the blue color of the river Isar, which is a non-vegetated area and should thus reach zero values. This suggested that the VHGPR model was better adapted to interpret water bodies as non-vegetated surfaces than the SNAP NN models.

## 4 Discussion

With the ambition to simplify the mapping of a variety of vegetation traits from satellite data, a prototype hybrid retrieval processing strategy was developed. The core idea of this strategy is that the retrieval models can be directly applied to TOA reflectance data, thus without the need for an atmospheric correction. In the following, the performances of the retrievals from S2 BOA (L2A) and TOA (L1C) data (Section 4.1), the variable-specific retrieval differences (Section 4.2), the comparison with the SNAP NN model reference vegetation products (Section 4.3), the advantages and limitations of the VHGPR models used (Section 4.4) and the RTMs (Section 4.5), and finally, future challenges and possible improvements of the workflow (Section 4.6) are discussed.

### 4.1 Performance of BOA and TOA Retrievals

The feasibility of retrieving key biophysical and biochemical variables from TOA radiance data has been earlier theoretically justified by means of a GSA of coupled RTMs. In a few studies, all input variables of the coupled leaf-canopy-atmosphere RTMs were entered into a GSA [50,78,79]. At the TOA scale, sensitivity results indicated that leaf biochemical and structural canopy variables predominantly drive radiance along the optical spectral range outside water vapor absorption. When applying the GSA over the full spectral range at 1 nm [50], the leaf variable *C_ab_* has the main effect in the visible region (VIS, 380−700 nm), and *C_w_* mainly affects the SWIR. The most dominant canopy structural variable in the VIS and SWIR optical domains is the LAI with a total sensitivity of more than 80%, although it must be remarked that in SAIL-like RTMs, the canopy structure is only defined by the LAI and average leaf angle. In more advanced RTMs, the role of canopy structure is spread over multiple variables, e.g., as demonstrated with INFORM [80].

Although no GSA was applied in our study, interestingly, the obtained variable retrieval accuracy from the BOA and TOA levels (see Table 3) was consistent with the GSA results. Furthermore, when validating against in situ reference data collected during the MNI campaigns, the same meaningful retrievals emerged. Overall, theoretical and validation results suggested that vegetation variables could be retrieved from TOA data with moderate to high accuracy. Moreover, we observed a slight trend of improved estimates from the TOA scale, in particular for crop traits at the canopy level (i.e., LAI and laiCw). Likewise, the former study of Estévez et al. [34] found that LAI retrieval at the TOA scale outperformed those at the BOA scale. It was argued that the quality of BOA data was influenced by the various processing steps involved to convert the L1C product into L2A, which further affected retrieval accuracy [81]. For instance, Figure 7 shows a few croplands exhibiting very high absolute uncertainty of leaf biochemical estimates from the BOA scale, which is no longer seen in estimates from TOA reflectance. Hence, potential uncertainty introduced in the processing steps may have been propagated to the estimates. Yet, the uncertainty patterns at both the BOA and TOA scales were generally alike, which suggested a similar prediction performance of the LAI at both scales (see Figure 6, bottom).

### 4.2 Variable-Specific Mapping

From space, crop traits at the canopy level (LAI, laiCab, laiCw) are usually more successfully estimated than leaf-level variables, e.g., *C_ab_* and *Cw*. That trend was strongly observed by our results for both L1C and L2A scales and was also confirmed by a few similar studies exploring Sentinel-2 data [40,82,83]. A possible explanation for poorer retrieval accuracy at the leaf level was given by Xie et al. [40], who argued about the compensation effects between LAI and *C_ab_* leading to the well-known ill-posed inverse problem. The retrieval accuracy of leaf biochemicals when derived from canopy reflectance may also be affected by the strength of the signal transmitted from the leaf to the canopy scale, i.e., signal dissemination, which is mainly controlled by structural traits such as the LAI or leaf angle distribution [84,85].

When using simulated data to evaluate the theoretical performance of the VHGPR models, the estimation of leaf-level traits works much better than with in situ data. This was also reflected by our results (theoretical performances vs. in situ) with NRMSE values around 13% vs. 29−36% for *C_ab_* and an NRMSE of 12% vs. 60% for *C_w_*. Except for the LAI, this difference was also found for canopy traits, but less pronounced. Higher NRMSE values obtained by in situ evaluation can be also explained by the few data available from the MNI campaigns, i.e., few samples collected on only two crop types. Obviously, more in situ data would lead to more pronounced trends. However, care is required; uncertainty introduced by the ground measurements themselves can also lead to poorer validation results. For example, in the case of *C_w_* measurements, leaf water may have been lost during the transport of samples from the field to the laboratory. Other sources of uncertainty were the S2 acquisitions and pre-processing: real-world data always include some artifacts, which may not be interpreted correctly by the retrieval model developed over the synthetic data base, hence leading to poorer predictions over unknown data.

To the the best of our knowledge, this was the first study estimating multiple crop traits from S2 L1C data using a hybrid retrieval approach. Though comparison studies at the TOA scale are lacking, our results can be compared at the scale of S2 L2A data, with a few recent works assessing the retrieval of leaf and canopy chlorophyll content and the LAI. For instance, Darvishzadeh et al. [85] estimated leaf chlorophyll content in spruce stands with RTM inversion (applying an optimized S2 band setting) with an RMSE of 8.1 μg/cm^2^, an NRMSE of 33%, and an R^2^ of 0.36, which was less accurate than our in situ validation (RMSE = 6.5 μg/cm^2^, NRMSE = 29%, R^2^ = 0.47). Our results also outperformed those of the study of Xie et al. [40], who obtained an R^2^ of 0.68 and an RMSE of 0.94 m^2^/m^2^ for the LAI of winter wheat (the same held true for the *C_ab_* and laiCab retrievals). Furthermore, Ali et al. [86] estimated laiCab from S2 data and compared empirical and RTM inversion methods with the best results obtained by partial least squares regression (PLSR) with an R^2^ of 0.78 and an RMSE of 0.22 g/m^2^. Here, this variable was retrieved with higher precision in terms of R^2^ (0.85), but not in absolute measures (RMSE = 0.38 g/m^2^). Still, comparing the results of different studies was not straightforward. First, the conditions of those studies were completely different, including the retrieval methods, types of vegetation, or number and magnitude of in situ reference data. Second, the use of only one or two different goodness-of-fit statistical indicators limited the comparability between studies. For instance, the RMSE is strongly influenced by the magnitude of trait values, whereas the NRMSE is sensitive to outliers. Hence, an optimal set of evaluation statistics should be used comprised of at least three or four measures of different statistical categories for appropriate and valid comparison of the results obtained by different studies (see also Richter et al. [87] for a discussion on this topic).

### 4.3 Comparison against SNAP Vegetation Products

The SNAP NN models for the estimation of crop traits are considered as reference products and have been also evaluated by a few studies [40,76,77,88]. For instance, Kganyago et al. [88] compared the retrieval performance of SNAP-derived LAI with existing global LAI products. They found only moderate consistency between different products (RMSE of 0.5−0.6 m^2^/m^2^), which was a lower match than VHGPR versus the SNAP NN models of our study with an LAI-RMSE of 0.49 m^2^/m^2^ (BOA) and an LAI-RMSE of 0.4 m^2^/m^2^ (TOA; see Figure 8, left). Still, the SNAP NN model strongly overestimated the LAI values at the MNI site at this point of time (beginning of July), with unrealistic LAI values up to 11 at the BOA scale. These results were consistent with the findings of Estévez et al. [34], where the SNAP NN model suffered from extreme LAI overestimation at the BOA, but less at the TOA scale. This tendency was also observed for laiCab and laiCw, but not for FVC (see Figure 9): a comparison between VHGPR and SNAP NN estimates at the TOA scale revealed lower discrepancies than at the BOA scale. The strong overestimation of the higher laiCab (>2 g/m^2^) and laiCw (>1000 g/m^2^) values by SNAP NN compared to the VHGPR models (Figure 8, middle plots) was most likely also influenced by the high LAI predictions. Our findings were confirmed by a laiCab retrieval study [86], indicating the tendency of overestimation by SNAP NN models from Sentinel-2 data (for forests).

In contrast to ours and other studies reporting retrieval inconsistencies, Pasqualotto et al. [76] found relatively good accuracy when estimating LAI and laiCab with the SNAP NN models. The authors even suggested that the generated biophysical products offered a great potential for hybrid retrieval workflows within agricultural applications. Though this may be certainly valid, caution is required when interpreting the LAI (and other canopy-level traits) estimates by the SNAP NN model in the later (mature) growing season. Using TOA instead of BOA reflectance for retrieval could be one solution to mitigate this problem. Further, we proposed to test our workflow as an alternative, since the implemented VHGPR models were not particularly prone to overestimating canopy-level traits. Generally, our results may be of specific relevance since we are not aware of any other study providing a comparison of multiple crop traits’ retrieval between SNAP NN BOA and TOA products, except for the LAI [34,88].

### 4.4 Machine Learning Regression Model and Uncertainty

A key aspect of the hybrid workflow was the choice of the machine learning regression algorithm. We favored the VHGPR algorithm in hybrid designs, as the family of Gaussian processes achieved generally superior performances compared to other MLRAs in vegetation properties’ estimation, e.g., [27,30,75,89]. VHGPR yielded also high performances of LAI estimation in our previous study at both the BOA and TOA scales [34] and provided meaningful uncertainty estimates. The systematic superiority of VHGPR over standard GPR was explained by its feature of assuming the variance of the noise process as dependent on the signal, hence letting the noise power vary smoothly throughout the input space [32]. Once the VHGPR model is established, it can be applied to any S2 L1C or L2A image for mapping diverse leaf biochemical compounds and canopy biophysical crop traits, as demonstrated here over the German MNI agricultural site. Consistency among TOA and BOA retrievals and the uncertainty pattern, in particular for canopy traits (i.e., FVC, LAI, laiCab, and laiCw), confirms the potential of VHGPR models for implementation into EO retrieval processing chains.

The majority of existing methods for the estimation of vegetation biochemical and biophysical traits from EO data only provide point predictions. This is, however, unfavorable, since those estimates can be affected by uncertainty in the model parameterization or input data [90]. With the provision of uncertainty maps, we overcame this drawback. For instance, these maps could function as spatial masks using a defined threshold (e.g., of 20%, as proposed by the Global Climate Observing System (GCOS) [91]) in order to exclude samples or improve mapping. In this context, the spectra of bare soils or senescent crop fields, which cannot be appropriately modeled by the RTM used, could be extracted from the scene and added to the training data to improve robustness and accuracy [92]. Moreover, the information of per-pixel uncertainty can be used to assess the portability of the established models over spatial and temporal scales [93]. Note that the provided uncertainty intervals by the Gaussian process models mainly cover the error sources of the retrieval algorithm including the parameterizations and simplifications of the RTMs used. There are two more broad clusters of uncertainty sources in the process of estimating vegetation traits from Earth observation data [94]: (i) sensor design, data acquisition, and pre-processing; and (ii) uncertainty arising from field data collection. It should be kept in mind that these additional error sources are not covered by VHGPR uncertainty maps.

### 4.5 Advantage and Limitations of the RTMs Used

TOA observations above a cropped surface are basically influenced by three main factors: (1) atmospheric conditions, (2) leaf and canopy characteristics, and (3) soil background [53]. Accurate prediction of crop traits from satellite sensors, such as S2, depends on reliable models, able to accurately describe each of these three components in the atmosphere-surface system [51]. In our study, we used the well-known coupled leaf optical properties and canopy reflectance model PROSAIL and the atmosphere model 6SV, both of which have proven to be suitable for satellite applications providing reasonable accuracy for diverse applications (see [37,38] for an overview of PROSAIL applications and [58,60] for 6SV applications). An overall advantage of these RTMs is their simplicity, which facilitates the whole workflow: relatively low computational times go along with good to high accuracy in predicting most biophysical and biochemical crop traits of interest. Though both are 1D turbid medium approaches, the scattering and absorption processes within the atmosphere and canopy are accurately described by these models taking into account the underlying physics. The limited number of variables further facilitates parameterization. On the other hand, oversimplification is also a limitation, leading to the increase of uncertainty in forward modeling.

The idea to combine vegetation RTMs with atmospheric RTMs in forward modeling was initiated almost two decades ago by Verhoef and Bach [53,95], who coupled the SLCmodel with MODTRANto generate TOA observed radiance. This coupled leaf-canopy-atmosphere approach was further elaborated for vegetation trait mapping applications from air- and space-borne imaging spectroscopy data by Laurent et al. [43–45]. More recently, Mousivand et al. [47] and Verhoef et al. [96] estimated vegetation traits from TOA radiance combining the SCOPE, global soil vectors (GSVs), and MODTRAN5 models. In these two studies, the MODTRAN atmospheric input parameters were assumed as known and used to simulate the optical coefficients for the translation of TOC to TOA reflectance. Finally, only surface characteristics were retrieved. Hence, to further improve this research line, the soil-plant-atmosphere radiative transfer (SPART) model was introduced [52] and was recently applied for the retrieval of vegetation properties from Sentinel-3 OLCITOA radiance [97]. SPART is a combination of three models for soil reflectance, canopy, and atmosphere radiative transfer. As a specificity, this model assumes the surface to display non-Lambertian reflectance behavior. Indeed, vegetation canopies are not optimal Lambertian diffusers. Uncompensated atmospheric scattering caused by the Lambertian assumption may systematically introduce uncertainty into the retrieval results. The magnitude of biases increases with enhanced scattering in the atmosphere caused by higher aerosol concentrations [98]. On the other hand, assuming a Lambertian approximation renders the computation more feasible by reducing the required size of the simulated data sets used for training MLRAs. Moreover, according to the studies of Thome et al. [99], Settle et al. [100], and Wang et al. [98], the error caused by Lambertian approximation only introduces a small error in the final product retrieval (albedo) for near-nadir observations where the observation geometry is not in the hot spot direction.

### 4.6 Future Challenges and Possible Improvements of the Workflow

Further improvements of the TOC modeling concept could be achieved when moving towards more complex (3D) vegetation models, e.g., DART [101]. These 3D canopy models describe the mechanistic link between the vegetation properties and the radiation regime within heterogeneous crop canopies more accurately compared to the 1D models used here. Apart from synthetic vegetation spectra, also the collection of non-vegetated spectra in the training data set can be broadened to make the model more generally applicable, e.g., by making use of spectral libraries. At the TOA scale, the implementation of more accurate atmosphere RTMs, e.g., libRadtran [56], may also improve the retrieval accuracy. This notwithstanding, the usage of more complex RTMs at both canopy and atmosphere scales increases the theoretical uncertainty through a higher number of input variables if no prior knowledge is available [94]. A high number of input variables also challenges the development of regression models: enlarging the size of the training data set goes along with increasing computational costs, in particular for kernel-based algorithms, such as VHGPR. In order to optimize training data sets in terms of size and diversity, smart sample reduction strategies, also known as active learning (AL), will be tested within a future study [25,102,103]. To our knowledge, AL heuristics have not yet been applied over coupled vegetation-atmosphere states as proposed by a recent survey [104]. This could provide an efficient solution in view of developing cost-efficient kernel-based machine learning regression models within operational S2 TOA retrieval workflows. Furthermore, future studies will be dedicated to TOA retrieval workflows of vegetation traits from other sensors, in particular using data from recently launched and upcoming spaceborne imaging spectroscopy missions.

As a final remark, it must be emphasized that this comparison exercise between our proposed modeling approach and the SNAP NN serves merely for demonstration purposes, i.e., as a proof-of-concept that TOA-based vegetation traits’ retrieval can be easily achieved, e.g., by developing models with the ALG-ARTMO software framework. The usage of S2 data served perfectly as a benchmark because of having good quality BOA and TOA products at disposal. Yet, in principle, the models can be trained for any type of TOA radiance, as long as these data were acquired under clear-sky conditions. The software framework can be freely downloaded at artmotoolbox.com/, (accessed on 15 April 2021). Both the SNAP NN and the VHGPR retrieval approaches presented here have their strengths and limitations, and the final decision about a method strongly depends on the users’ needs and software requirements.

## 5 Conclusions

In this study, we presented a computationally efficient approach of directly retrieving multiple crop traits from TOA Sentinel-2 reflectance data. To achieve this, a hybrid retrieval workflow was proposed, combining leaf-canopy-atmosphere RTMs and developing prototype retrieval models with VHGPR algorithms. The validation of the VHGPR retrieval models led to reasonably accurate results against theoretical and in situ reference data from an agricultural region. Moreover, whole images could be processed within a semi-automated processing framework. The evaluation of VHGPR retrieval models for six vegetation variables at both the S2 BOA and TOA scales led to the following main findings:

- Consistent theoretical performances at the BOA and TOA scales were achieved, suggesting that hybrid retrieval models can be directly applied to TOA radiance or reflectance data.
- The validation results and associated uncertainties suggested higher fidelity of the TOA model performances as opposed to the BOA.
- Canopy variables were more successfully retrieved than leaf variables.
- VHGPR models provided higher plausibility than the SNAP NN models for deriving vegetation products.

All in all, the results showed that vegetation (crop) traits’ estimation can be achieved directly from TOA reflectance, which suggests that atmospheric correction is not strictly necessary given a clear sky. Direct TOA-based retrieval not only simplifies the processing chain, but also helps to avoid potential uncertainty propagated by this step. Moreover, the capability of VHGPR models to generate uncertainty intervals enables verifying the portability of the models in space and time and can eventually support decision-making processes for agricultural applications. In summary, the presented retrieval workflow opens the door to monitoring biochemical and biophysical crop characteristics over agricultural areas within operational processing schemes based on Copernicus Sentinel-2 data products. Beyond S2, the workflow can be applied and tested to any reflectance or radiance images for mapping multiple crop traits using the ALG-ARTMO software framework.

## Figures and Tables

**Table 1:** Dates of MNI in situ data collection, S2 L1C/L2A acquisitions, crop type (ww: winter wheat), measured values of biochemicals: leaf chlorophyll content (*C_a_b*) and leaf water content (*C*_*w*_), and biophysical variables: leaf area index (LAI), canopy chlorophyll content (laiCab), and crop water content (laiCw).

MNI Date	S2 Date	Crop	*C_ab_* (μg/cm^2^)	*C_w_* (cm)	LAI (m^2^/m^2^)	laiCab (g/m^2^)	laiCw (g/m^2^)
21 April 2017	24 April 2017	ww	44.68	0.020	3.60	1.61	703.70
04 April 2018	07 April 2018	ww	51.95	0.020	0.28	0.15	55.98
18 April 2018	19 April 2018	ww	50.47	0.019	3.10	1.56	586.88
13 June 2017	13 June 2017	maize	38.45	0.015	0.21	0.08	32.14
26 June 2017	26 June 2017	maize	49.60	0.012	1.57	0.78	193.70
06 July 2017	06 July 2017	maize	51.03	0.014	2.88	1.47	416.36
09 August 2017	05 August 2017	maize	52.22	0.016	3.90	2.04	640.25
30 August 2017	25 August 2017	maize	53.67	0.017	3.06	1.64	510.28
03 July 2018	01 July 2018	maize	55.67	0.020	3.59	1.99	729.28
26 July 2018	31 July 2018	maize	60.79	0.025	3.61	2.19	891.00
08 August 2018	12 August 2018	maize	60.54	0.022	3.67	2.22	798.50
17 August 2018	17 August 2018	maize	57.40	0.022	3.39	1.94	734.81
22 August 2018	22 August 2018	maize	57.50	0.022	3.25	1.87	713.49
29 August 2018	27 August 2018	maize	55.07	0.020	3.83	2.11	754.18

**Table 2:** Parameterization of leaf (PROSPECT-4), canopy (4SAIL), and atmosphere (6SV) models, with the notations, units, ranges, and distributions of inputs used to establish BOA and TOA synthetic reflectance databases. $\bar{x}$: mean, SD: standard deviation. LHS: Latin hypercube sampling.

Model Variables		Units	Range	Distribution
*Leaf variables:* PROSPECT-4				
*N*	Leaf structure parameter	unitless	1.3−2.5	Uniform
*C_ab_*	Leaf chlorophyll content	(μg/cm^2^)	5−75	Gaussian ($\bar{x}$: 35, SD: 30)
*C_m_*	Leaf dry matter content	(g/cm2)	0.001−0.03	Gaussian ($\bar{x}$: 0.005, SD: 0.001)
*C_w_*	Leaf water content	(cm)	0.002−0.05	Gaussian ($\bar{x}$: 0.02, SD: 0.01)
*Canopy variables:* 4SAIL				
LAI	Leaf area index	(m^2^/m^2^)	0.1−7	Gaussian ($\bar{x}$: 3, SD: 2)
*α_soil_*	Soil scaling factor	unitless	0−1	Uniform
ALA	Average leaf angle	(°)	40−70	Uniform
HotS	Hot spot parameter	(m/m)	0.01	-
skyl	Diffuse incoming solar radiation	(fraction)	0.05	-
FVC	Fractional vegetation cover	(fraction)	0.05−1	-
*Atmospheric variables*: 6SV				
*O3C*	O_3_ column concentration	(amt-cm)	0.25−0.35	LHS
*CWV*	Columnar water vapor	(g · cm^−2)^	0.4−4.5	LHS
*AOT*	Aerosol optical thickness	unitless	0.05−0.5	LHS
*ALPHA*	Angstrom coefficient	unitless	0.05−2	LHS
*G*	Henyey−Greenstein asymmetry factor	unitless	0.6−1	LHS
*Illumination*/*observation conditions*: 4SAIL and 6SV				
*θ_s_*	Sun zenith angle	(°)	20−30	Uniform
*θ_v_*	View zenith angle	(°)	0	-
*ø*	Sun-sensor azimuth angle	(°)	0	-

**Table 3:** Theoretical k-fold (5k) cross-validation goodness-of-fit results of studied vegetation traits for the BOA and TOA. Units of the RMSE for *C_ab_* in μg/cm^2^, for *C*_*w*_ in cm, for LAI in m^2^/m^2^, and for laiCab and laiCw in g/m^2^. NRMSE in %. Variable abbreviations can be found in Table 1.

Variable	*C_ab_*		*C_W_*		FVC		LAI		laiCab		laiCw	
Level	BOA	TOA	BOA	TOA	BOA	TOA	BOA	TOA	BOA	TOA	BOA	TOA
**RMSE**	9.66	10.20	0.0063	0.0059	0.0589	0.0539	0.81	0.80	0.34	0.34	172.00	167.00
**NRMSE**	12.90	13.64	12.73	11.92	5.94	5.45	11.61	11.41	7.15	7.12	5.52	5.72
**R** ^2^	0.76	0.73	0.56	0.60	0.95	0.96	0.77	0.78	0.85	0.84	0.86	0.87

**Table 4:** Comparison results of BOA vs. TOA uncertainty maps for several crop traits. The RMSE, NRMSE, and R^2^ are given for the standard deviation (SD) maps (Figure 7) and the coefficient of variation (CV) maps (not shown).

Variable	*C_ab_*		*C_w_*		FVC		LAI		laiCab		laiCw	
Uncertainty Type	SD	CV	SD	CV	SD	CV	SD	CV	SD	CV	SD	CV
**RMSE**	4.04	19.09	0.0015	16.42	0.0113	21.94	0.0786	14.12	0.0362	12.31	26.68	15.54
**NRMSE (%)**	13.49	19.09	14.45	16.42	3.67	21.94	2.80	14.12	1.63	12.31	1.33	15.54
**R** ^2^	0.03	0.41	0.20	0.34	0.17	0.28	0.93	0.37	0.91	0.68	0.91	0.51

## Data Availability

Not applicable.
